# Open the Rings to Close the Cycle: The Complete Degradation of Riboflavin Returns Simple Building Blocks Back to Nature

**DOI:** 10.1021/acscentsci.5c02116

**Published:** 2025-11-21

**Authors:** Max Bernstein Sosa, Ikuro Abe

**Affiliations:** Graduate School of Pharmaceutical Sciences, 13143 The University of Tokyo, Tokyo 113-0033, Japan

## Abstract

The complete biochemical degradation of riboflavin converts a vital enzyme cofactor back into simple carbon and nitrogen sources.

## Graphical abstract

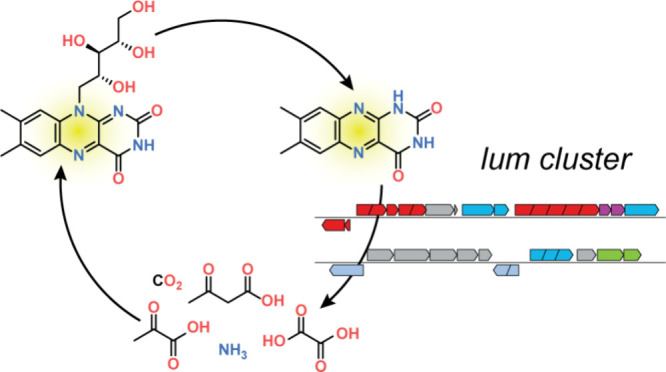

Metabolism is defined by the biochemical reactions which build (anabolism) and degrade (catabolism) chemical compounds. Enzymes are the primary catalysts of metabolism, but many of them rely on complex organic cofactors to facilitate otherwise inaccessible modes of reactivity. Flavoenzymes are proteins which use the vitamin B_2_-derived cofactors flavin adenine dinucleotide (FAD) or flavin mononucleotide (FMN) to catalyze a diverse set of, generally, redox reactions (Figure 1). These include dehydrogenation, hydroxylation, Baeyer–Villiger oxidation, and halogenation reactions; however, other reaction classes, as well as redox neutral reactions, have also been reported.^1^ The ability of flavin to act as either a one- or two-electron donor enables it to play key roles in electron transfer reactions, providing one-electron reducing equivalents to various metalloenzymes after receiving electrons from a two-electron hydride donor, such as a nicotinamide cofactor. More recently, flavoenzymes have been adopted for photobiocatalytic reactions, and extensive synthetic methodologies are being developed using this coenzyme.^2^

**Figure 1:**
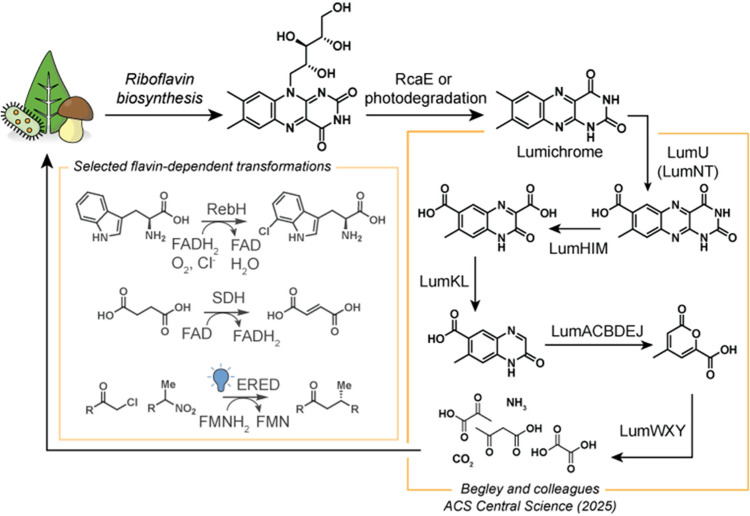
Riboflavin is biosynthesized by plants or microorganisms, and the flavins FAD or FMN can be used by various flavin-dependent enzymes. Degradation into lumichrome provides a substrate for the Lum pathway, described by Begley and co-workers, which converts lumichrome into simple organic acids, carbon dioxide, and ammonia. These simple metabolites can be reused by various organisms for growth and energy production.

Riboflavin biosynthesis is limited to plants, fungi, and microorganisms, but all organisms must mature riboflavin into FMN or FAD before it can be used by FAD or FMN-dependent flavoenzymes (Figure 1).^3^ While the biosynthesis of many cofactors is well understood, it remains unclear how these ubiquitous biomolecules are recycled into simple metabolic building blocks. For riboflavin, the only biochemically characterized step in its degradation is its separation into the ribose and lumichrome, a poorly soluble heterocycle.^4^ Despite the poor aqueous solubility of lumichrome, it has been shown to act as a potent chemical signaling molecule in both soil and marine environments, inducing metamorphosis in ascidians and various physiological responses in plants.^5,6^ There is also evidence that lumichrome interacts with transcriptional regulators, intercepting quorum sensing pathways and inducing biofilm formation in microorganisms.^7^ Perhaps, because of the low solubility of lumichrome, these interactions are operative even at low nanomolar concentrations.

In this issue of *ACS Central Science*, Begley and co-workers tackle an outstanding question in vitamin metabolism, describing a complete degradation pathway for lumichrome into ammonia, carbon dioxide, and simple organic acids (Figure 1).^8^ The *lum* cluster contains the set of genes responsible for encoding the lumichrome catabolizing enzymes. The first step in this pathway is catalyzed by LumU, a cytochrome P450 which improves the solubility of lumichrome through successive oxidations of a benzylic methyl group to the carboxylic acid. Then, hydrolytic degradation of the pyrimidine ring is performed by the sequential action of LumH, LumI, and LumM. This results in the net conversion of 7-carboxylumichrome into two molecules of ammonia, one molecule of carbon dioxide, and a quinoxaline that is an order of magnitude more soluble than lumichrome.

Riboflavin biosynthesis is limited to plants, fungi, and microorganisms, but all organisms must mature riboflavin into FMN or FAD before it can be used by FAD or FMN-dependent flavoenzymes (Figure 1). While the biosynthesis of many cofactors is well understood, it remains unclear how these ubiquitous biomolecules are recycled into simple metabolic building blocks.

The quinoxaline contains an α-imino acid-like motif, which is electronically similar to an α-keto acid. As α-keto acids are commonly decarboxylated by thiamine pyrophosphate (TPP)-dependent enzymes, the authors reasoned that at least one of the two TPP-dependent enzymes encoded in the cluster might be responsible for decarboxylation of this substrate. Unexpectedly, the enzymes LumK and LumL appear to function as a heterodimeric TPP-dependent decarboxylase. The resulting imine is then oxygenated by the molybdoprotein LumJ, producing a biologically rare oxamide motif.

At this point, the multicomponent Rieske dioxygenase catalyzes dihydroxylation of the substrate, leading to ring opening of the cyclic oxamide. The hydrolytically labile oxamide nonenzymatically decomposes into a carboxylic acid and ammonia. Oxidation by the extradiol dioxygenase leads to ring opening, decarboxylation, and recyclization to a pyrone with release of the two-carbon oxamic acid. This product can be further hydrolyzed into ammonia and oxalic acid. Extradiol dioxygenases are commonly found in catabolic pathways of aromatic compounds, and these pathways are well-characterized. The pyrone is then degraded by a series of hydrolytic enzymes, converting it into pyruvic acid and acetoacetic acid, thus completing the degradative pathway of lumichrome.

While this pathway describes the complete conversion of lumichrome into simple carbon and nitrogen sources, many questions remain unanswered. For example, LumKL is proposed to form a heterooligomeric enzyme that performs a TPP-dependent decarboxylation of a heterocyclic imino acid substrate. Biophysical or structural characterization of this complex may provide insights into why this specific class of substrates can be utilized by LumKL, perhaps leading to new developments in the biodegradation of environmental pollutants or in biocatalysis. Heterodimeric TPP-dependent decarboxylases are rare, so comparison of this system with the other known natural heteromeric TPP enzymes may unveil common patterns in the evolution of these systems. Also, it is unclear how lumichrome is initially accessed by the P450 LumU since the substrate faces solubility issues. Is there a possibility that various enzymes in this pathway are secreted into the environment prior to forming a more readily bioavailable substrate, or is lumichrome directly imported from the environment? Simple understanding of the kinetic parameters of the lumichrome degradation enzymes should also be addressed. As lumichrome can act as a signaling molecule at low nanomolar concentrations, would improved solubility of the substrate even be necessary if the catabolic pathway also functioned efficiently at low substrate concentrations?^6^

LumN and LumT are NAD-dependent oxidases encoded by the cluster which can oxidize the first P450 oxidation product from an alcohol to an acid. But if the P450 LumU is sufficient to perform the sequential oxidation of the methyl group to a carboxylic acid, why are these enzymes needed? Perhaps biochemical redundancy is an evolutionary solution to pathway-level substrate scope expansion.

Additionally, the pathway appears to encode enzymes which perform redundant reactions. LumN and LumT are NAD-dependent oxidases encoded by the cluster which can oxidize the first P450 oxidation product from an alcohol to an acid. But if the P450 LumU is sufficient to perform the sequential oxidation of the methyl group to a carboxylic acid, why are these enzymes needed? Perhaps biochemical redundancy is an evolutionary solution to pathway-level substrate scope expansion. Modified lumichromes derived from naturally occurring flavin derivatives, such as 6-hydroxy FMN, 8-formyl FMN, or prenyl FMN, might also be metabolized by this pathway, and alternative enzymes could be required to accommodate them.^9^ Further work will be needed to explain this biochemical redundancy.

As lumichrome is found throughout soil environments, further research on the evolution of this gene cluster and the ecology of lumichrome catabolism in soil ecosystems could shed light on some microbial ecology. This direction is especially important considering lumichrome’s role as a potent plant signaling molecule excreted by plant-associated microbes, as well as its ability to engage with bacterial quorum sensing pathways. Does the richness of *lum* cluster-harboring actinobacteria correlate with the abundance of lumichrome producers? Can the presence of lumichrome catabolizing species prevent quorum signaling-induced biofilm formation in bacterial competitors? By examining the distribution of metabolic pathways in a given metagenome, perhaps researchers can further contextualize the ecological relationships between coexisting organisms.

Overall, this work represents an important body of knowledge which illustrates how a ubiquitous metabolite can be broken down into simple sources of carbon and nitrogen. Complete reconstitution of the enzymatic activity of the minimum set of enzymes required for lumichrome biodegradation provides a platform for studying the mechanisms of these proteins. Moreover, engineering and importing this pathway into heterologous hosts could enable the creation of strains useful for bioremediation or the degradation of heterocyclic pollutants. Further, it opens new questions regarding the ecology of lumichrome-utilizing bacteria and how they may shape various interspecies interactions in the soil environment.
